# The Potential Impact of the Gut Microbiota on Neonatal Brain Development and Adverse Health Outcomes

**DOI:** 10.3390/children11050552

**Published:** 2024-05-05

**Authors:** Maria Tzitiridou-Chatzopoulou, Jannis Kountouras, Georgia Zournatzidou

**Affiliations:** 1 School of Healthcare Sciences, Midwifery Department, University of Western Macedonia, Koila, 50 100 Kozani, Greece; mtzitiridou@uowm.gr; 2 Second Medical Clinic, School of Medicine, Ippokration Hospital, Aristotle University of Thessaloniki, 54 642 Thessaloniki, Greece; jannis@auth.gr; 3 Department of Business Administration, University of Western Macedonia, 50 100 Kozani, Greece; 4 Department of Accounting and Finance, Hellenic Mediterranean University, 71 410 Heraklion, Greece

**Keywords:** gut–brain axis microbiome, neonatal, neuroscience, functional gastrointestinal disorders

## Abstract

Over the past decade, microbiome research has significantly expanded in both scope and volume, leading to the development of new models and treatments targeting the gut–brain axis to mitigate the effects of various disorders. Related research suggests that interventions during the critical period from birth to three years old may yield the greatest benefits. Investigating the substantial link between the gut and brain during this crucial developmental phase raises fundamental issues about the role of microorganisms in human health and brain development. This underscores the importance of focusing on the prevention rather than the treatment of neurodevelopmental and neuropsychiatric disorders. The present review examines the gut microbiota from birth to age 3, with a particular focus on its potential relationship with neurodevelopment. This review emphasizes the immunological mechanisms underlying this relationship. Additionally, the study investigates the impact of the microbiome on cognitive development and neurobehavioral issues such as anxiety and autism. Importantly, it highlights the need to integrate mechanistic studies of animal models with epidemiological research across diverse cultures to better understand the role of a healthy microbiome in early life and the implications of dysbiosis. Furthermore, this review summarizes factors contributing to the transmission of gut microbiome-targeted therapies and their effects on neurodevelopment. Recent studies on environmental toxins known to impact neurodevelopment are also reviewed, exploring whether the microbiota may mitigate or modulate these effects.

## 1. Introduction

The microbiota colonizes a newborn’s body at a crucial time when the brain is undergoing significant developmental processes. In mice, this process involves the infiltration of the brain by microglia and the deliberate elimination of growing neurons. In the early postnatal period of mice, there is a substantial rise in the number of microglia, which are immune cells found in the brain. Additionally, these microglia experience alterations in their physical structure and gene expression [1]. In mice, the first week after birth is characterized by a high concentration of cell death. This process, known as apoptosis, destroys around 50% of the neurons that have stopped dividing [2]. A study conducted by Mosley et al. (2017) revealed that there are sudden and significant alterations in neuronal cell death after birth [3]. This finding prompted us to propose the hypothesis that the colonization of bacteria at birth can influence and shape this process [4,5].

For instance, germ-free (GF) mice demonstrate that a lack of microbiota at birth is linked to specific alterations in cell death and an increase in microglial labeling in the hippocampus and hypothalamus [4,6]. The observed impacts did not manifest throughout the prenatal period, but rather emerged during a timeframe of 12 to 14 h after delivery. This indicates that immediate contact with microorganisms is a crucial factor. Additionally, we discovered that animals born with a microbiome had significantly elevated levels of pro-inflammatory cytokines, such as interleukin 1β and tumor necrosis factor α, in their brains compared to mice born without a microbiota. According to a recent study by Hoffiz et al. (2021), it was discovered that neurons in the paraventricular nucleus (PVN) began to exhibit activity three hours after birth [6]. The PVN is a crucial component of the brain that is responsible for receiving input from the vagus nerve and playing a vital role in regulating both the immune system and stress response. The timing of this occurrence aligns with the introduction of microbiota into the gastrointestinal tract of the infant. However, a causal relationship between the reasons that can contribute to the transmission of the gut microbiome to neonates during the immediate peripartum period and how this can affect infants has not yet been investigated.

Therefore, this review aims to investigate exposure to microbes during the immediate peripartum period. Our focus is on addressing important questions and identifying areas where our understanding is lacking. Specifically, we aim to thoroughly examine the factors that contribute to the transmission of the gut microbiome to neonates. Additionally, we will highlight the current research trends regarding its potential health effects on infants. Following the above, the aim of the current study is twofold: (i) to examine deeply the factors that contribute to the transmission of the gut microbiome to neonates, and (ii) to highlight the research trends regarding the health impact that it may have on infants’ brains. The current study has developed a bibliometric analysis based on R statistical programming language, utilizing the bibliometric tools of Biblioshiny (Version 4.2.0) and VOSviewer (Version 1.6.19) to achieve its objective. For the purpose of the bibliometric analysis, 845 research documents (research articles) were retrieved from Scopus and analyzed with the bibliometrix formula of R statistical language. The research primarily explores the relationship between brain development, neurodevelopmental psychiatric illnesses, and the gut microbiota. The results of this study demonstrate the impact of the gut microbiota and early life events on the neurobehavioral development of preterm newborns. The findings suggest that the accumulation of acute traumatic and upsetting events might adversely impact neurobehavioral development outcomes.

The organization of this study is as follows. Section 2 provides an overview of the existing literature, Section 3 explains the materials and methods applied to address the study topic, and Section 4 presents the analysis. Section 5 of this research paper provides a discussion on the findings and limitations. Additionally, Section 5 serves as the conclusion of the report.

## 2. Literature Review

The traditional dichotomy between “environmental factors” and “genetic factors” is no longer sufficient, given the microbial connection between the mother and the newborn. Early life initiates this microbial association when the infant’s mucosal and epidermal surfaces colonize with the maternal microbiota, a collection of bacteria from the mother’s body. The potential outcomes of this initial exposure to microorganisms include the establishment of a mutually beneficial relationship between the host organism and the microbiota, which may influence juvenile development and maintain the balance between health and illness [7,8,9]. 

The microbiota plays a crucial role in maturation and development. In addition to regulating infant health during infancy, the microbiota also contributes to long-term well-being. The optimal development and maturation of the microbiota occur between implantation and early childhood. Infants can influence their microbiota despite genetic and environmental predispositions. Despite the recent surge in understanding the role of microorganisms in human disease, the complex nature of the human condition remains a significant challenge to the practical application of this knowledge. The microbiota of neonates remains a subject of constant debate and controversy. Previously, the presence of bacterial, viral, and fungal organisms originating from the vagina was attributed to intrauterine conditions affecting the embryo [10,11,12]. Nevertheless, scientific advancements have revealed that the fetus’s health could potentially be affected by the microbiota present in the oral cavity and gastrointestinal tract, which can be transmitted via the circulatory system.

The microbiomes of the mother’s oral cavity, vagina, and intestines undergo alterations during pregnancy. These changes are influenced by variables such as nutrition, medication usage, pathogens, tension, and host genomes. Derbyshire and Gray (2014) state that the vaginal microbiota (VM) of expectant women is more stable than that of non-pregnant women, with Lactobacillus species being the most prevalent. Recent years have disproved the previously held belief that the uterus lacked sterility. The biomass of the endometrium microbiota is finite in quantity [13]. Unfortunately, there is still a lack of comprehensive understanding regarding the endometrial microbiota, and its potential implications for fetal development and pregnancy outcomes remain unclear [14]. 

Pregnant women exhibit comparable levels of gut microbiota (GM) diversity and consistency to those of non-pregnant women; however, their microbiota is more dispersed and has distinct compositions. The diminished variety of genetically modified organisms (GMs) negatively impacts the well-being of both the fetus and the mother, particularly during pregnancies marked by complications. For example, chronically hypertensive rodents experience disturbances in GM remodeling during pregnancy. 

### Early Brain Development: An Additional Potential for the Transmissions of Microbiota

The involvement of the GM in the brain’s initial development provides an additional potential mechanism for microbiota transmission. Recent research has provided substantial evidence supporting the notion that bacteria actively participate in the development and progression of the central nervous system (CNS). The recognition of GMs’ roles in various neurodevelopmental processes has become increasingly evident. These processes include myelination, the development of the blood–brain barrier (BBB), neurogenesis, and the maturation of microglia, all of which play crucial roles in regulating animal cognition and behavior [15]. Neuronal cell development and optimal functioning of the growing body require a variety of nutrients that are released by the gut. Moreover, recent studies have suggested that the GM may directly enhance brain development processes, resulting in long-lasting health benefits. 

Cautery endothelial cells, pericytes, astrocytes, and tight junction proteins make up the blood–brain barrier (BBB). During early development in the womb, a barrier forms to separate the brain from the rest of the body’s circulation, thereby restricting the flow of substances between them. Furthermore, it facilitates the transport of essential nutrients and chemicals that are critical for the brain’s efficient functioning and maintenance. For the BBB to function properly, it requires the presence of metabolites produced by microorganisms, such as short-chain fatty acids (SCFAs), as well as a well-balanced GM [16]. According to Mollgaard and Saunders (1986), the permeability of the BBB diminishes as sterile fetuses approach maturity [17]. The BBB is less effective in germ-free (GF) mice because occludin and claudin-5 are not expressed as much in the brain’s endothelial layer. This lets more macromolecules pass through. Scientists have discovered that administering or gently contacting butyrate, a type of SCFA produced by the GM through food fermentation, may reduce blood flow to the brain in mice lacking a GM.

Furthermore, neurogenesis, the process by which new functioning neurons are formed, may play a role in the transfer of genetic material throughout the brain development of newborns. This process occurs through neural stem and progenitor cell differentiation. Neurogenesis and neuronal plasticity are essential for learning, memory, cognition, and stress response and are particularly abundant in the hippocampus, which is the hub for cognitive activities. Sarubbo et al. (2022) explain how maintaining a balanced microbiota in the colon might influence the microenvironment, leading to the promotion of neuronal growth [18]. According to recent research comparing mice without a GM (GF mice) and mice with a normal GM (SPF mice), several substances produced by the GM may pass through the placenta and into the developing baby, affecting and influencing its growth and development. Peptidoglycan (PG), a component of the bacterial cell wall, can cross the placenta and enter the infant brain. It activates Toll-like receptor 2, resulting in an increase in the expression of FOXG1, a crucial transcription factor that controls the process of development and neurogenesis. As a result, neuron growth occurs in the forebrain. Salvo et al. (2020) recently published a study showing a new connection between the impact of cytokines generated by microbiota-regulated microbial activity and the process of neurogenesis [19].

In addition, the GM may indirectly influence neural plasticity in the CNS by regulating the maturation and movement of neurons via the ephrin B and reelin pathways. Ephrin B is essential for preserving the integrity of the gut epithelial barrier, whereas the GM also controls reelin, a membrane glycoprotein that aids in neural migration [20].

Increasing data suggest that neurotrophins and neurotransmitters produced by the GM may interact with intricate survival and differentiation pathways in various regions of the brain to alter the fate of neural stem cells. There is a link between the growth and maturation of synapses and the plasticity and maturity of neurons. Administering neonatal prebiotics (BGOs) to mice at 22 days of age resulted in elevated levels of synaptophysin and brain-derived neurotrophic factor (BDNF) in the hippocampus, as compared to other prebiotics [21,22]. Both BDNF and synaptic vesicle protein synaptophysin act as signaling molecules that facilitate the formation of synapses between neurons [23]. They also play a crucial role in the survival, growth, maturation, and maintenance of different populations of brain cells.

The GM releases serotonin, a neurotransmitter and signaling chemical, into the gut lumen, promoting the growth of new neurons in adults. Moreover, research has shown that the GM exhibits a crucial impact on serotonin signaling pathways, affecting both the digestive system and several regions of the brain. Various studies have also presented data about the influence of the GM on the control of adult neurogenesis. According to the findings, labeling cells that were undergoing growth resulted in a higher level of adult dorsal hippocampus neurogenesis in bromo-deoxyuridine GF mice as compared to mice that were allowed to develop naturally. The observed phenotypic state remained unchanged, despite the presence of microbes. The results of this study suggest that a lack of microorganisms leads to an unusual increase in adult dorsal hippocampus neurogenesis. Moreover, the research discovered that microbial signals had an impact on the formation of new neurons in the hippocampus throughout the crucial period of early life [24,25].

Moreover, research links immunosuppression, which modifies the GM’s composition, to a decrease in neurogenesis. The specific molecular mechanism by which the microbiota and its related metabolites control adult neurogenesis remains unclear. Liu et al. (2022) propose that neuroinflammatory processes, along with humoral and metabolic pathways, may play a role in mediating this process [26]. Recent results in several disciplines suggest that neuroinflammatory pathways may indeed be involved. The presence of microorganisms in the intestines promotes the maintenance of a healthy enteric neural system in adult rats by triggering the growth of new nerve cells via the activation of Toll-like receptor 2.

In addition, O’Leary et al. (2018) discovered a reduction in the messenger RNA (mRNA) levels of BDNF across the entire hippocampus [27]. The reduction in viability and neuronal growth described earlier was particularly pronounced in animals which had undergone vagotomies. Furthermore, the microbiome indirectly influences hippocampus neurogenesis by regulating the neural immune system. The investigation demonstrated that administration of dextran sodium sulfate induced acute inflammation in the colons of mice. This inflammation was linked to a problem with the GM, higher levels of cytokines linked to T-helper 17 cells and pattern recognition receptors, and the presence of ionized calcium-binding adaptor protein 1, which shows that microglial cells are active. Concurrent with these changes, there was a decrease in the rate of adult hippocampus neurogenesis, leading to the development of behavioral impairments.

Compared to mice kept in groups, isolation causes a decrease in IL-6 and IL-10 levels in the hippocampus and neurogenesis. This is like the way that insufficient social engagement throughout early life might weaken the integrity of the GM. Diminished hippocampus neurogenesis is closely associated with anxiety, depressive-like behaviors, learning impairment, neuroinflammation, and other symptoms. In addition, Liu et al. (2022) reveal a clear connection between these symptoms and changes in the structure of the GM.

## 3. Materials and Methods

We conducted the bibliometric analysis using the Biblioshiny web interface of Bibliometric R. Researchers can effectively conduct a wide range of scientometric analyses by leveraging its robust and integrative capabilities. These capabilities include data import and conversion, filtration, and the creation of a vast array of analytics and plots for multiple levels of metrics. We imported the cleaned dataset into Biblioshiny and generated descriptive statistics, such as author productivity and citations by country and affiliation. The bibliometrix function biblioNetwork generated a graphical network diagram that illustrates the authors’ collaborative efforts. We applied stringent criteria to co-authorships in the network, mandating a minimum of ten collaborations. We also used VOSviewer software to visualize the findings related to co-occurrence and co-authorship analyses [28,29].

Descriptive statistics were acquired by utilizing the bibliometrix program, which is an integral part of the R package. We compared the proportions of pre-registered and guideline-compliant studies published in ophthalmology and non-ophthalmology journals using Pearson’s Chi-squared test [30,31]. The R programming language and the open-source RStudio software (version 2023.03.0-daily+82.pro2)were exclusively utilized for all statistical analyses and data visualizations. Our analysis classified a *p*-value as statistically significant if it was below 0.05. The data for this study were obtained from the Scopus database by employing keywords, including neonatal, gut–brain axis, intestinal microbiome, neuroscience, and gut microbiome. The methodical explanation of the keyword exploration procedure is displayed in Table 1.

**Figure 1:**
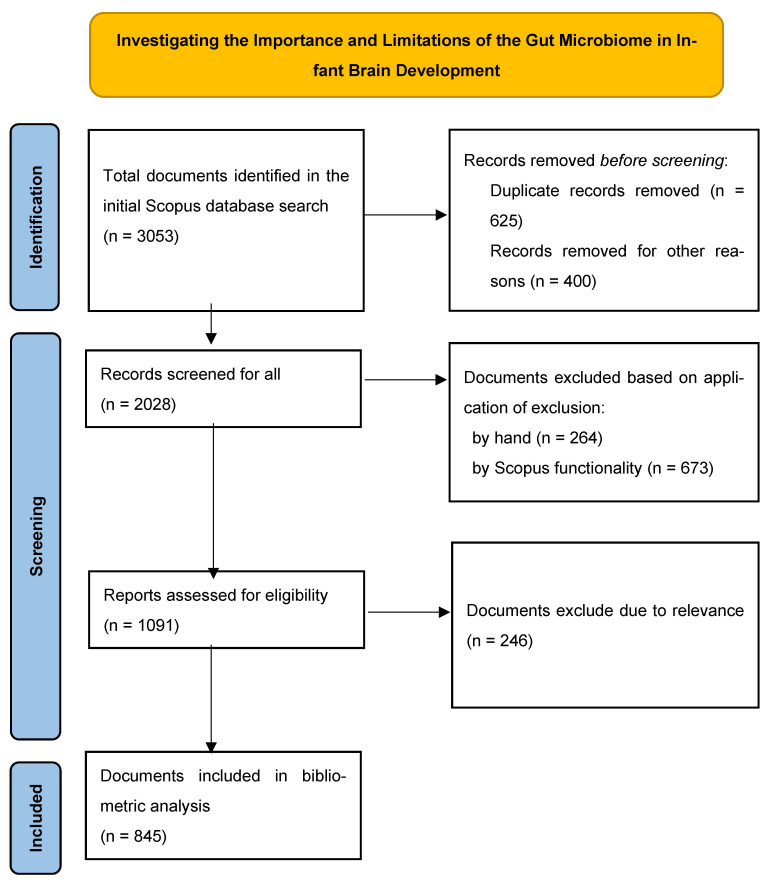
PRISMA flow diagram: article selection for bibliometric analysis.

Furthermore, the PRISMA flow diagram (Figure 1) provides a visual representation of the crucial stages involved in the selection of a reliable set of articles for bibliometric analysis [32,33,34]. The first search yielded a cumulative total of 3053 sources, as per the database. By narrowing the selection of articles, the total number was reduced to 2028. Subsequently, a thorough examination of 1091 publications was conducted to exclude those that were unnecessary or had scopes that were too wide, making them inappropriate for this study project. The aim of this research was to determine the variables that impact the transfer of the gut microbiota to newborns and the resulting health effects on children. After thoroughly analyzing the chosen research articles, we noticed that several sources did not clearly specify the features and qualities of the area under investigation in their titles or keywords. The search criteria were modified to include only publications that were directly relevant to the current study subject while excluding irrelevant references. Following the implementation of the filtering procedure, a total of 845 scientific papers remained. Furthermore, the following are the inclusion and exclusion criteria, as outlined in the PRISMA method, which was employed to aid in the source selection process: (i) the publication date, (ii) the health condition associated with the transmission of the gut microbiota and its effect on infant brain development, (iii) the language of publication (English-only research studies were considered), and (iv) geographic considerations, including specific regions, states, countries, or populations. Afterwards, each of these papers was included in the bibliometric analysis.

## 4. Results

### 4.1. Network Analysis

Table 2 displays the journals that garnered the greatest quantity of research submissions pertaining to the topic of study from 2014 to 2023. The table showcases the most relevant sources based on the quantity of publications. Throughout the research period, the field that generated the highest number of relevant publications (40 articles) was nutrients. Moreover, when it came to examining the mechanisms that lead to the transmission of the gut microbiome and its impact on newborns, the *International Journal of Molecular Sciences* held the second position, with a total of twenty (20) papers only focused on this topic. The journals *Frontiers in Microbiology* and *Frontiers in Neuroscience* together had published a total of fourteen (14) research articles pertaining to the topic. In addition, *Neuroscience and Biobehavioral Reviews*, which had already published thirteen (13) publications, was one of the top five most significant sources in the area.

According to Table 2, the main subjects covered in the journals were immunology, microbiology, and neurology. This research demonstrates a robust correlation between neurology and the subject under investigation. The evidence suggests that the GM may influence brain function and cognitive development via the production of hormones, immunological agents, and metabolites. This highlights a crucial element of the research, indicating that modifying the gut microbiota might enhance or address neurological problems. In addition, the Scopus and Scimago databases included all the chosen journals. The subject being examined had an h-index of about 157, indicating that scholarly articles in this area obtain an average of more than 157 citations. This picture highlights the crucial significance and wide-ranging influence of studies on the processes behind the transmission of GM substances to neonates and their major influence on brain development.

Table 3 presents the studies with the highest numbers of citations in the field of investigating factors impacting the transfer of the GM and its consequences on infants. Among all the research conducted on the topic, the study titled “The Microbiota-Gut-Brain Axis” had received the most citations. This work underscores the growing focus on understanding the molecular and physiological basis of psychiatric, neurodevelopmental, age-related, and neurodegenerative diseases influenced by the microbiome. The GM and the brain communicate through various routes, including the immune system, tryptophan metabolism, the vagus nerve, and the enteric nervous system. This communication involves the exchange of microbial metabolites, such as the mentioned SCFAs, short-chain fatty acids, branched-chain amino acids, and peptidoglycans. Several factors may affect the microbiota’s composition during early life, including infection, mode of birth, antibiotic use, dietary patterns, environmental pressures, and host genetic characteristics. Microbial diversity decreases with age. Stress significantly impacts the microbiota–gut–brain axis across all life stages. Recent research has demonstrated a robust correlation between the GM and various disorders, including autism, anxiety, obesity, schizophrenia, Parkinson’s disease, and Alzheimer’s disease. Animal models have been instrumental in elucidating the connection between the regulation of fundamental brain functions, such as neurogenesis and myelination, and microbiome-induced microglia activation. Moreover, ongoing translational human research will greatly advance the field, focusing on understanding the mechanisms underlying the relationship between the microbiota, stomach, and brain. The aim is to gain a more comprehensive understanding of how microorganisms can be leveraged to develop treatments and therapies for neuropsychiatric disorders [35,36]. 

The article titled “What is the Healthy Gut Microbiota Composition? A Changing Ecosystem across Age, Environment, Diet, and Diseases” ranked third among the most-cited documents. This study primarily comprises a literature review aiming to provide a comprehensive summary of the research on the balance of the GM within individuals and across populations. It emphasizes the significant mutualistic relationship between changes in the GM and the development of illness. GM dysregulation is associated not only with intestinal problems but also with various extra-intestinal diseases, including metabolic and neurological disorders. Investigating the causes or consequences of these imbalances in the GM in relation to both health and illness would be beneficial. Additionally, understanding how to preserve or restore a healthy composition of the GM could facilitate the development of effective therapeutic interventions.

Table 4 details the primary affiliations of researchers who had published articles examining the factors contributing to the transmission of the gastrointestinal microbiome to neonates and its effects on infants. The large number of published papers (236 publications) in this field highlights Irish academic institutions as frontrunners. University College Cork stands out prominently in Ireland for its distinguished research contributions, particularly on infant GM transmission. Specifically, the APC Microbiome Ireland SFI Research Centre, affiliated with this university, is widely recognized as a leading research institute specializing in microbiome studies [7,47,48,49,50]. Established in 2003, the Alimentary Pharmabiotic Centre fosters collaboration among researchers from diverse fields. The APC has cultivated a vibrant multidisciplinary environment, facilitating collaboration between doctors, clinician-scientists, and basic scientists, thereby encouraging the exchange of ideas and resources. The study of the GI bacterial population, known as the GM, is a rapidly expanding field with implications for various areas of human medical and veterinary research as well as for the economic well-being of society. The microbiota plays a crucial role in disease treatment and prevention, as well as in providing beneficial food components and potential medications. Additionally, it offers a wealth of unique biomarkers that may signal the likelihood of certain diseases. 

Researchers at the APC actively pursue collaborations with organizations in many industries, including both major and small businesses, local enterprises, and global corporations. They have vast knowledge and experience in the fields of food, agriculture, medicines, biotechnology, and diagnostics. The APC’s organizational structure is characterized by a grid-like configuration of vertical pillars or intellectual research domains, which are bolstered by collaborative technology platforms. This framework promotes the creation of groundbreaking concepts, inspires a profound thirst for knowledge, and expedites research that is in line with the demands of the industry. The APC, as a national institution and resource, promotes change by creating a creative atmosphere, questioning obsolete concepts in education, and actively cooperating with worldwide partners in academia and industry. APC Microbiome Ireland builds upon its previous accomplishments and anticipates the future with passion, vigor, and assurance.

**Figure 2:**
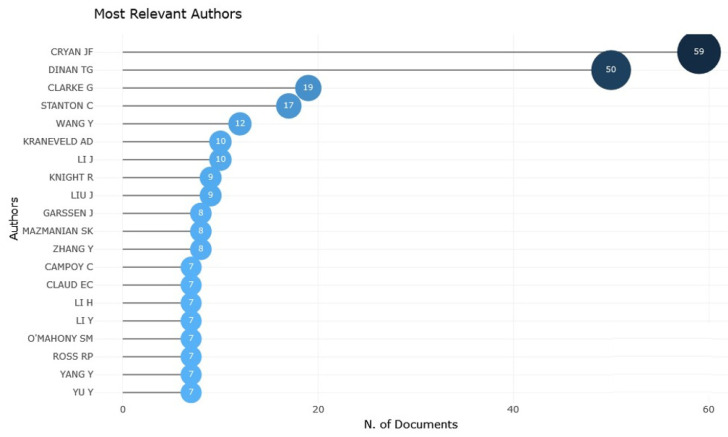
Most relevant authors. Source: Biblioshiny.

Based on previous findings regarding Irish universities and their significant contributions to research, John F. Cryan, a professor and chair of the Department of Anatomy and Neuroscience at University College Cork, emerged as one of the most influential authors in the field of investigating factors related to the transmission of the GM to infants (Figure 2). He has published 59 papers on this topic and serves as the principal investigator at the APC Microbiome Institute. Professor Cryan’s ongoing research focuses on understanding the interplay between the brain, gut, and microbiome and its relevance to stress-, psychiatric-, and immune-related illnesses across critical periods of an individual’s life. With over 600 scholarly papers and book chapters to his credit, which were subject to rigorous peer review, Professor Cryan boasts an H-index of 146, as measured by Google Scholar in October 2022. Additionally, he holds the position of senior editor for the journals *Neuropharmacology* and *Neurobiology of Stress*.

Furthermore, Dr. Timothy Dinan, an esteemed professor of psychiatry and principal investigator at the Alimentary Pharmabiotic Centre at the University of Cork, Ireland, holds a prominent position as the medical director of the Atlantia Food Clinical Trials. His 50 publications establish him as one of the most influential scientists in his field of study. Dr. Dinan is a distinguished scientist, specializing in researching the immunological and endocrine aspects of depression, particularly examining how the GM affects stress-related illnesses. Moreover, he possesses extensive knowledge in the fields of intestinal health, drug studies, and the nervous system’s role in emotional disorders, among other areas within psychiatry. His work has had a substantial impact on both sides of the Atlantic, as evidenced by his prestigious positions as a fellow of the Royal Colleges of Physicians and Psychiatrists as well as a fellow of the American College of Physicians. In 1995, Dr. Dinan received the Melvin Ramsey Prize for his groundbreaking research on stress biology. His projects have received funding from such organizations as Science Foundation Ireland, the Health Project Board, and the European Union. According to Expertscape, he ranked as the top global expert on the microbiota in 2019 and was recognized as one of the top 100 global makers and mavericks.

### 4.2. Bibliometric Analysis

**Figure 3:**
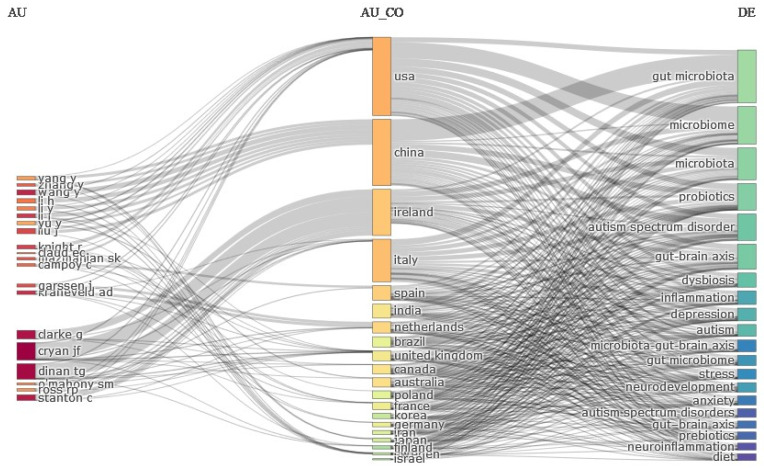
Three-field plot. Source: Biblioshiny.

We generated a three-field plot, also known as a Sankey diagram, to illustrate the distribution of research subjects across different countries and the temporal relevance of the cited articles. The design consisted of three fields: authors, country, and keyword. Figure 3 shows that the main thing that US researchers have been examining when they study the GM and how it gets passed on to newborns is how it affects babies’ brain development, especially when it comes to autism spectrum disorder (ASD). ASD is a neurodevelopmental condition characterized by impairments in typical brain development. The recent discovery of the microbiota–gut–brain axis signifies the reciprocal relationship between the brain and gut, implying that it has the potential to impact numerous neurological disorders, including autism. Most patients with autism experience gastrointestinal (GI) symptoms. Numerous studies have demonstrated the substantial effects of early colonization, mode of administration, and antibiotic use on the GM and the onset of autism.

In addition, based on the previous section and the findings presented in Figure 2, the mentioned Professor John F. Cryan, recognized as the leading researcher in the global research community, and Figure 3 confirm his specific focus on studying the factors contributing to the development of the GM and dysbiosis in children. The GM, which dwells in the human GI system, plays a crucial role in maintaining the health of the host. New developments in next-generation sequencing methods have shown that dysbiosis, an imbalance in the normal gut microbiota, may cause many illnesses by disrupting the symbiotic relationship between the host and the beneficial bacteria. The GM undergoes a significant shift from birth until the age of three, becoming like an adult’s GM. Developing a well-balanced GM during infancy is crucial since any imbalance might persist throughout adulthood, leading to dysbiosis [25,51,52]. Subsequently, many studies have focused on determining the factors that impact the composition of the GM in infants. Furthermore, the plot illustrates how stress and depression can alter the composition of the GM through inflammation, stress hormones, and autonomic changes. GI microorganisms subsequently secrete neurohormones, metabolites, and toxins that have the potential to modify both mood and feeding patterns. Certain species of bacteria may promote a dysregulated appetite.

**Figure 4:**
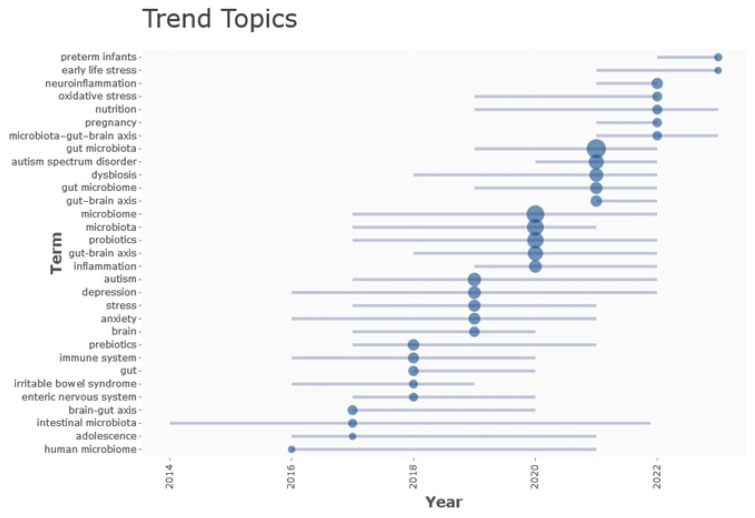
Trend topics. Source: Biblioshiny.

Moreover, Figure 4 confirms the significance of anxiety and stress factors in the transmission of the gut microbiome to infants. Specifically, the figure illustrates the growing body of research investigating the transmission of adverse effects to progeny that is caused by maternal stress, anxiety, melancholy, and inflammation. A contemporary subject of inquiry pertains to the “brain-gut axis”, which describes how the brain instructs and influences immune and neurological health via bidirectional communication with the microbiome, comprising trillions of bacteria, fungi, and viruses that inhabit the gastrointestinal tract [53,54,55,56]. Systemic dysfunction, including altered immune responses, metabolic function, and neurodevelopment, has been linked to alterations in the composition of the microbiome’s inhabitants or changes in the structure of the microbial community, which are induced by external stimuli, such as antibiotics, infections, and diet [4,57,58]. Both animal and human models have shown this to occur. The mother primarily transmits microorganisms to her infants through the birth canal, skin-to-skin contact, and breastfeeding. The mother is the greatest source of microorganisms for her children. The composition of the maternal microbiome directly influences the microbial species present in the progeny. Therefore, these amounts correlate with the health outcomes of the offspring. In addition to altered metagenome, metabolome, and/or immune responses that may impact host processes, a perturbed microbiome may subsequently influence health through aberrant metabolism of dietary intake [59]. Thus, it is crucial for the development of the progeny that the mother maintains a consortium of health-promoting microorganisms. Regrettably, the presence of psychiatric disorders and inflammation can disrupt the structure of microbial communities, potentially leading to adverse effects on the health of offspring. This is evident from research examining the impact of antibiotic usage by mothers during pregnancy on the offspring microbiome, which has implicated the transfer of the maternal microbiome into the offspring. The microbiome, immunity, and social behavior of progeny have all been involved in maternal stressor exposure according to rodent research.

Furthermore, the results shown in Figure 4 highlight the robust correlation between the GM and early life stress, which might potentially impact preterm newborns. Preterm babies are particularly susceptible to both dysbiosis in the GM and neurological damage. Although the connection between early dysbiosis and short-term clinical outcomes is well recognized, the association with long-term infant health has only recently received attention. During the early stages of life, there is a substantial convergence in the time periods when the muscular system and the neurological system experience growth and development. Animal models first revealed the correlation between the GM and neurodevelopment. Nevertheless, in the last ten years, an increasing amount of research has recognized that GM characteristics are likely to be one of the factors that influence the development of neurological and mental problems in humans. 

**Figure 5:**
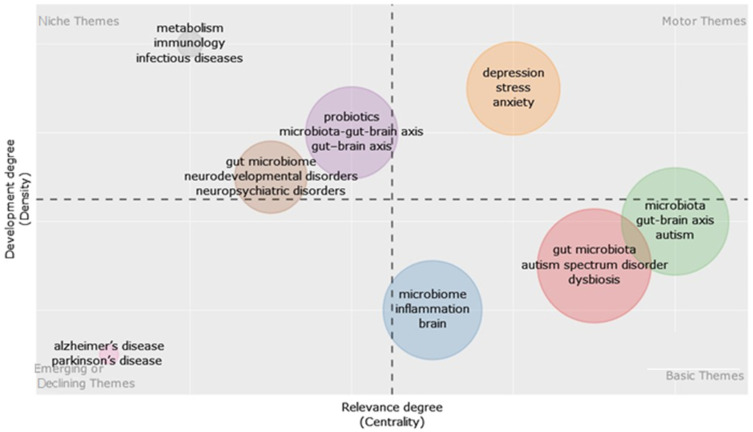
Thematic map. Source: Biblioshiny.

Furthermore, the present research incorporates a thematic map of the inquiry into the elements that contribute to the transfer of the GM to newborns for further evaluation. The purpose of creating a thematic map is to gain a better understanding of the area’s current condition and potential for long-term survival. The aim of this research is to provide academics with information on potential avenues for further investigation into certain thematic areas related to the GM in newborns and its impact on them. Thematic analysis involves collecting ideas from clusters of keywords used by authors and scrutinizing the interconnections between these notions. These motifs vary in their distinct features, namely their density and centrality. The x-axis reflects centrality, whereas the y-axis represents density. Centrality quantifies the extent of interconnection between various topics, whereas density assesses the amount of cohesiveness among the nodes. The degree to which specific topics are developed and have value is determined by two key traits: development and significance. Establishing more connections with other nodes enhances a node’s centrality, significance, and position within a thematic network. The degree of coherence among nodes in a research subject reflects its density and regulates its capacity to grow and maintain itself. Figure 5 shows a thematic map of the domains of smart learning environments. We split it into four quadrants, specifically named Q1 to Q4.

The Q1 quadrant represents motor themes; the Q4 quadrant represents basic themes; the Q2 quadrant represents niche themes; and the Q3 quadrant represents emerging or declining themes. The graph clearly demonstrates the correlation between NPDs and the GM in babies. Changes in the composition and diversity of the GM have been specifically associated with various mental illnesses, including anxiety, major depressive disorder (MDD), bipolar disorder (BD), Parkinson’s disease (PD), schizophrenia, attention-deficit hyperactivity disorder (ADHD), and other neurodegenerative conditions. These diseases are characterized by deviations in brain functioning that lead to disruptions in behavior, cognition, and emotion. A closer analysis revealed the association between these diseases and neuromodulatory chemicals like serotonin and dopamine, as well as malfunctions in the brain networks that control cognition and emotions. Accumulating research suggests that the microbiome, namely the GI environment, has a major influence on the mental well-being of people. While it is well acknowledged that there is a gut–brain communication axis, current research suggests that its formation and control are influenced by the GM.

**Figure 6:**
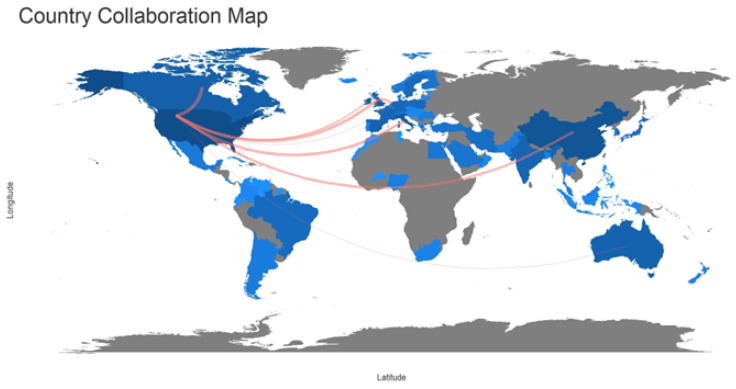
Country collaboration map. Source: Biblioshiny.

Finally, Figure 6 illustrates international collaborations. The azure color on the diagram represents international collaboration in research. The crimson border connecting the states denotes the degree of collaboration among the authors. It is intriguing to observe which nations have participated in such collaborations regarding the factors that contribute to the transmission of the intestinal microbiome to neonates, as evidenced by their high number of publications in this area. Collaboration can lead to policy exchange and market cooperation. However, it is notable that the United States, China, Germany, and the United Kingdom have engaged in the most significant partnerships with countries that are at times geographically distant from one another. As a result, they have failed to develop the theme to its full potential.

## 5. Discussion and Conclusions

Scholars have devoted considerable attention to the complex interplay between the GI tract and the CNS, which includes the potential influence of gut-dwelling microorganisms on this relationship. Understanding the relationship between the GM and the developing brain of early infancy remains largely unknown since most previous research has primarily concentrated on adult subjects and has established connections using animal models. It is indisputable that the GM plays a vital role in neurodevelopment, as demonstrated using germ-free rodents [50,51,53,54,55,56]. Immunological deficiencies and severe neurodevelopmental abnormalities ensue when the intestines of rodents fail to undergo typical colonization during their early developmental stages. Behavioral issues, such as challenges with learning and memory, are also caused by this. 

Notable anatomical and physiological variations observed in germ-free animals include significantly underdeveloped hippocampi, cortices, striata, and cerebella. In addition, their striatal turnover of dopamine is diminished, whereas microglia function is impaired, and serotonin and norepinephrine turnover are increased and decreased, respectively [57,58]. The CNS and the GM exchange information via various pathways. Activation directly activates the vagus nerve, which connects the enteric nervous system to the CNS. Moreover, a multitude of metabolites generated by the microbiome find their way into the bloodstream via the intestinal barrier [59,60]. Transmitting across the BBB, these metabolites can modulate neurological processes. In addition to microbially associated molecular patterns (MAMPs) and metabolites, the microbiome is capable of transmitting signals to the immune system. Neurotransmitters, including gamma-aminobutyric acid (GABA), dopamine, noradrenaline, and histamine, are also synthesized by the GM. These neurotransmitters intrinsically link to the development and operation of the nervous system. The colon, its microbiome, and the CNS establish connections through the hypothalamic–pituitary–adrenal axis (HPA axis), enteric and autonomic nervous systems, and hormone signaling. Microbial metabolites regulate these associations regularly; the systemic immune system frequently opposes these metabolites. The relationship between the stomach and the brain, particularly in relation to anxiety and stress disorders, has been associated with the HPA axis.

The cytokines released by immune cells, particularly microglia and astrocytes, exert a significant influence over neurophysiology. Microglia, for instance, safeguard the brain across all life stages by producing cytokines, engaging in phagocytosis, and stimulating immune responses [61,62]. They also play pivotal roles in synaptic transmission regulation, neural circuit formation, and pruning during early development, with research indicating their susceptibility to signals from the microbiome. Furthermore, factors such as age and gender may influence microglia’s responsiveness to microbiome cues. Astrocytes, which are similar to microglia, are needed for the CNS to work because they control the flow of nutrients and keep the BBB strong. Microbiota metabolites can change how these cells perform their functions.

Understanding the impact of the gastrointestinal microbiota and its metabolites on early neurodevelopment is a burgeoning area of research. While investigations into the gut–brain axis have typically focused on neurodegenerative processes, evidence suggests that microbial activity may also influence neurogenesis. Studies using germ-free mouse models have shown how the GM and its metabolites can affect the development of brain regions that are crucial for memory, like the hippocampus. Conversely, interventions such as probiotic administration in murine models have shown potential in mitigating these effects. Furthermore, animal models of early neurodevelopmental neuroinflammation have highlighted the role of TNF-α synthesis in impairing memory formation. Our research endeavors to elucidate the impact of the GM during the critical developmental window spanning from birth to three years old. This period, characterized by rapid growth, offers an opportune moment to optimize developmental outcomes [63,64,65]. Given the dynamic nature of the microbiome, which is susceptible to alterations, we are particularly eager to delve into the intricacies of the gut–brain axis during this pivotal phase.

Moreover, the research on the gut microbiome has specifically focused on ASD as a neurodevelopmental consequence. ASD, characterized by impaired social communication and repetitive behaviors, often manifests immune dysregulation in the GI tract. Common GI symptoms like diarrhea, constipation, and stomach discomfort are frequently correlated with the severity of behavioral impairments in ASD and are associated with microbial imbalances. Intriguingly, the etiology of ASD remains poorly understood and likely arises from a complex interplay of genetic and environmental factors. The microbiome serves as a nexus between external and internal influences, with the gut acting as a protective barrier. Genetic and environmental risk factors for ASD may directly impact the native microbiome. Unraveling the causal relationship in older children and adults with ASD poses challenges due to potential reverse causation. Various medical conditions linked to ASD, such as immunological dysfunction and gastrointestinal issues, may indirectly influence the microbiome [66,67]. Specifically, an imbalance in the GM, in conjunction with the immunological and GI symptoms observed in ASD, may influence the development of the nervous system, neuronal functioning, and the manifestation of atypical behaviors associated with ASD [66,67].

This research has some limitations. The primary limitation lies in the collection of sample data. Technological constraints related to the analysis software hindered the integration of data from multiple databases, making it an unfeasible approach. This study sourced its sample from the Scopus database, potentially excluding relevant data. Obtaining sample data from diverse independent databases would undoubtedly enhance the research. Furthermore, we recommend refining the search criteria to achieve greater precision. This limitation should incentivize further investigation, prompting scholars to explore techniques for data extraction from multiple databases using more generalized keywords to conduct a comprehensive inquiry. Overall, the objective of this research was to provide valuable insights into the current state of research and potential avenues for future investigation to academicians, particularly novice scholars in the field of infant gut microbiome.

## Figures and Tables

**Table 1:** Keyword search formula.

Step	Keyword Search
1	((“microbioma”) AND (“infants”))
2	((“microbioma” OR “microbiome”) AND (“infants” OR “neonatal”))
3	((“microbioma” OR “microbiome” OR “gut microbiome”) AND (“infants” OR “neonatal”))
4	((“microbioma” OR “microbiome” OR “gut microbiome”) AND (“infants” OR “neonatal”) AND (“gut brain axis”))
5	((“microbioma” OR “microbiome” OR “gut microbiome”) AND (“infants” OR “neonatal”) AND “gut brain axis” OR “brain development”))
6	((“microbioma” OR “microbiome” OR “gut microbiome”) AND (“infants” OR “neonatal”) AND “gut brain axis” OR “brain development”) AND (“neuroscience”))
7	((“microbioma” OR “microbiome” OR “gut microbiome”) AND (“infants” OR “neonatal”) AND “gut brain axis” OR “brain development”) AND (“neuroscience” OR “neurological disorder”))
8	((“microbioma” OR “microbiome” OR “gut microbiome”) AND (“infants” OR “neonatal”) AND “gut brain axis” OR “brain development”) AND (“neuroscience” OR “neurological disorder” OR “cognitive neuroscience”))
9	((“microbioma” OR “microbiome” OR “gut microbiome”) AND (“infants” OR “neonatal”) AND “gut brain axis” OR “brain development”) AND (“neuroscience” OR “neurological disorder” OR “cognitive neuroscience” OR “nutritional neuroscience”))
10	((“microbioma” OR “microbiome” OR “gut microbiome”) AND (“infants” OR “neonatal”) AND “gut brain axis” OR “brain development”) AND (“neuroscience” OR “neurological disorder” OR “cognitive neuroscience” OR “nutritional neuroscience”)) AND (LIMIT-TO (DOCTYPE, “ar”)) AND (LIMIT-TO (PUBSTAGE, “final”) OR LIMIT-TO (PUBSTAGE, “aip”)) AND (LIMIT-TO (SRCTYPE, “j”))

**Table 2:** Most relevant sources. Source: Biblioshiny.

Sources	Articles	Subject Area	H-Index	SJR List
*Nutrients*	40	Nursing	178	Q1
*International Journal of Molecular Sciences*	20	Biochemistry, Genetics, and Molecular Biology	230	Q1
*Frontiers i* *n Microbiology*	14	Immunology and Microbiology	201	Q1
*Frontiers i* *n Neuroscience*	14	Neuroscience	136	Q2
*Neuroscience a* *nd Biobehavioral Reviews*	13	Neuroscience	271	Q1
*Brain Behavior a* *nd Immunity*	12	Neuroscience	184	Q1
*Frontiers o* *n Psychiatry*	12	Psychiatry and Mental Health	96	Q1
*Frontiers i* *n Cellular a* *nd Infection Microbiology*	11	Immunology and Microbiology	105	Q1
*Microorganisms*	10	Immunology and Microbiology	66	Q2
*Gut Microbes*	9	Immunology and Microbiology	96	Q1

**Table 3:** Most globally cited documents. Source: Biblioshiny.

Paper	Total Citations	TC per Year
The Microbiota-Gut-Brain Axis [37]	2036	339
Dynamics and Stabilization of the Human Gut Microbiome during the First Year of Life [38]	1918	192
What is the Healthy Gut Microbiota Composition? A Changing Ecosystem across Age, Environment, Diet, and Diseases [39]	1615	269
Current understanding of the human microbiome [40]	1322	189
The Role of Short-Chain Fatty Acids From Gut Microbiota in Gut-Brain Communication [41]	1094	219
Gut/brain axis and the microbiota [42]	962	96
The Central Nervous System and the Gut Microbiome [43]	895	99
Signals from the gut microbiota to distant organs in physiology and disease [44]	877	97
Microbiota Transfer Therapy alters gut ecosystem and improves gastrointestinal and autism symptoms: an open-label study [45]	849	106
Microbial Reconstitution Reverses Maternal Diet-Induced Social and Synaptic Deficits in Offspring [46]	747	83

**Table 4:** Most relevant affiliations. Source: Biblioshiny.

Affiliations	Country	Articles
University College Cork	Ireland	256
Utrecht University	The Netherlands	53
University Of California	USA	49
Baylor College Of Medicine	USA	44
University Of Granada	Spain	40
Jiangnan University	China	31
Xuzhou Medical University	China	30
McMaster University	Canada	26
Nanjing Medical University	China	26
University Of California San Diego	USA	26
